# The Benefits of a Dental Operating Microscope for Tooth Extractions: A Case Report

**DOI:** 10.3390/dj13060243

**Published:** 2025-05-29

**Authors:** Bruno Calkovsky, Ladislava Slobodnikova, Sarah Kalmanova, Maria Janickova

**Affiliations:** Clinic of Stomatology and Maxillofacial Surgery, Jessenius Faculty of Medicine, University Hospital Martin, Comenius University in Martin, 03601 Martin, Slovakia; ladislava.slobodnikova@uniba.sk (L.S.); sarah.kalmanova@uniba.sk (S.K.); maria.janickova@uniba.sk (M.J.)

**Keywords:** operating microscope, atraumatic tooth extraction, dentoalveolar surgery, microsurgery

## Abstract

**Background/Objectives:** Operating microscopes are becoming increasingly common tools in dentistry and dentoalveolar surgery due to their ability to enhance procedural precision and control. This case report aims to highlight the benefits of the operating microscope in minimizing surgical trauma and improving clinical outcomes during tooth extractions. **Methods:** Three clinical cases involving potentially complicated tooth extractions were treated using a dental operating microscope. The procedures were performed without flap elevation, extensive bone removal, or suturing, with the goal of reducing trauma to adjacent structures. **Results:** In all three cases, the use of the microscope facilitated minimally invasive extractions. The surrounding tissues were preserved, and the patients experienced uneventful healing. Clinical re-evaluation was performed several weeks postoperatively to confirm mucosal healing and absence of complications. **Conclusions:** The dental operating microscope allowed for precise, minimally traumatic extractions, resulting in favorable healing outcomes. Although limited, current literature and the presented cases suggest promising results and superior outcomes when incorporating a microscope into dentoalveolar surgical practice. This case report further supports the role of magnification in improving surgical precision and patient care.

## 1. Introduction

Modern minimally invasive treatments that aim to preserve healthy tissue are only possible with magnification, and thus, magnifying devices are widely used in many medical fields [1]. In dentistry, the use of an operating microscope is common among endodontists. Gradually, specialists with a focus on conservative dentistry, fixed prosthetics, periapical surgery, or periodontology are becoming increasingly aware of the need for high magnification [2].

Contemporary dentistry, including dentoalveolar surgery, focuses on the minimally invasive and detailed treatment of patients. Magnification combined with intense lighting provides a better discrimination of contrasts, colours, and the structure of objects and allows for more detailed orientation in the operative field. In addition to overcoming the limitations of the human eye during minimally invasive treatments, an operating microscope allows dentists to work in an ergonomic position and reduce eye strain [3].

By using a dental operating microscope, it is possible to distinguish points as little as 0.006 mm apart, whereas the human eye can only distinguish two distinct points if they are at least 0.2 mm apart [4]. The main motivation for the technological development of magnifying devices was the limited size and depth of the operating field, insufficient lighting, and unsuitable working positions of physicians during procedures. Modern dental operating microscopes reach a light output of around 120,000 lux and offer 6–25 times magnification, which allow for the visualisation of microscopic details in the operative field [5].

A high degree of magnification is essential in endodontics for the identification and inspection of root canals [1]. In most cases, lower-degree magnification that approaches the levels of advanced magnifying loupes is fully sufficient for dentoalveolar surgery [6].

Coaxial lighting provides intense illumination of the working field without casting shadows that could optically modify the structures in the operating field. A powerful light source incorporated into a microscope facilitates the differentiation of tooth tissues from alveolar bone [7]. An operating microscope also allows, for example, the most apical parts of the extraction site to be inspected, which can be useful in identifying the remains of granulation tissues or extruded endodontic filling materials, checking sockets for the presence of tooth fragments or filling materials, or directly visualising suspected oroantral communication.

The limited number of studies [8,9] that have investigated the use of an operating microscope in dentoalveolar surgery have shown promising results and superior outcomes. The conclusions of a recent randomised controlled trial demonstrating the advantages of an operating microscope over dental loupes for extractions and alveolar ridge augmentation are particularly encouraging, especially due to the enhanced magnification and illumination. The aim of this case report was to expand upon the existing scarce literature data and to further demonstrate the advantages of the use of a microscope in dentoalveolar surgery.

## 2. Study Endpoints

The primary endpoint was uneventful healing, assessed through postoperative follow-ups. Secondary endpoints included the presence or absence of complications, the need for surgical interventions (e.g., flap elevation or bone removal), and the clinical utility of the operating microscope in simplifying the procedure. These outcomes were evaluated clinically at two time points (7–10 days and 4–6 weeks), and radiographs were used when clinically justified.

## 3. Case Report

### 3.1. Case 1

**Figure 1:**
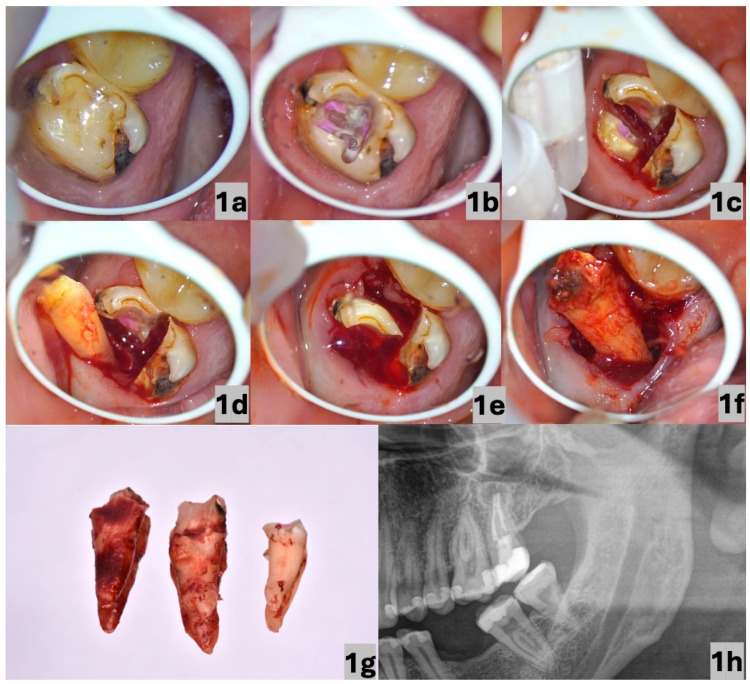
Occlusal view of the second left maxillary molar (secondary decay present) (**a**); identification of the entrances to the root canals (marked with a coloured composite) (**b**); separation of the roots based on the identified entrances to the root canals (**c**); elevation of the distobuccal root (**d**); elevation of the mesiobuccal root (**e**); elevation of the palatal root (**f**); extracted roots (**g**); and segment of panoramic radiograph showing the second left maxillary molar (**h**). The images in (**a**–**f**) were taken using an operating microscope under 6× magnification.

A 31-year-old woman without a clinically significant personal, medicinal, or allergic history visited the clinic with acute pain located in the maxillary left quadrant. She identified the second left maxillary molar as the source of the pain (Figure 1a). An examination revealed tenderness to percussion, the presence of secondary caries, and previous substandard endodontic treatment, which was visualised on a panoramic radiograph (Figure 1h). The patient refused re-endodontic treatment in combination with the necessary prosthetic rehabilitation of the tooth. She opted for an extraction.

Under infiltration anaesthesia comprising 1.7 mL of an articaine hydrochloride solution with adrenaline in a ratio of 1:100,000 (Septanest Forte, by Septodont, Saint-Maur-des-Fossés, France), we chose to separate the roots prior to their elevation. Both of these procedures were performed using an operating CJ-Optik Flexion Twin microscope (CJ-Optik GmbH & Co. KG, Asslar-Werdorf, Germany) due to the increased risk of a tooth fracture during the extraction. The procedure included the identification of the entrances to the root canals (Figure 1b), the separation of the roots (Figure 1c), and the subsequent elevation of the distobuccal, mesiobuccal, and palatal roots in the indicated order (Figure 1d–g) while a thorough debridement of the alveolar socket was performed. The performed Valsalva manoeuvre was negative, and due to the minimal trauma of the surrounding tissues, there was no need to suture the extraction wound. The patient was clinically re-evaluated 8 days postoperatively to assess soft tissue healing and to rule out complications such as dry socket or infection. A subsequent follow-up was conducted approximately 5 weeks after the procedure to confirm complete mucosal healing. Since no complications were observed, further radiographic follow-up was deemed unnecessary at that moment. Wound healing was considered uneventful, with no need for antibiotic therapy or the prolonged use of analgesics.

### 3.2. Case 2

**Figure 2:**
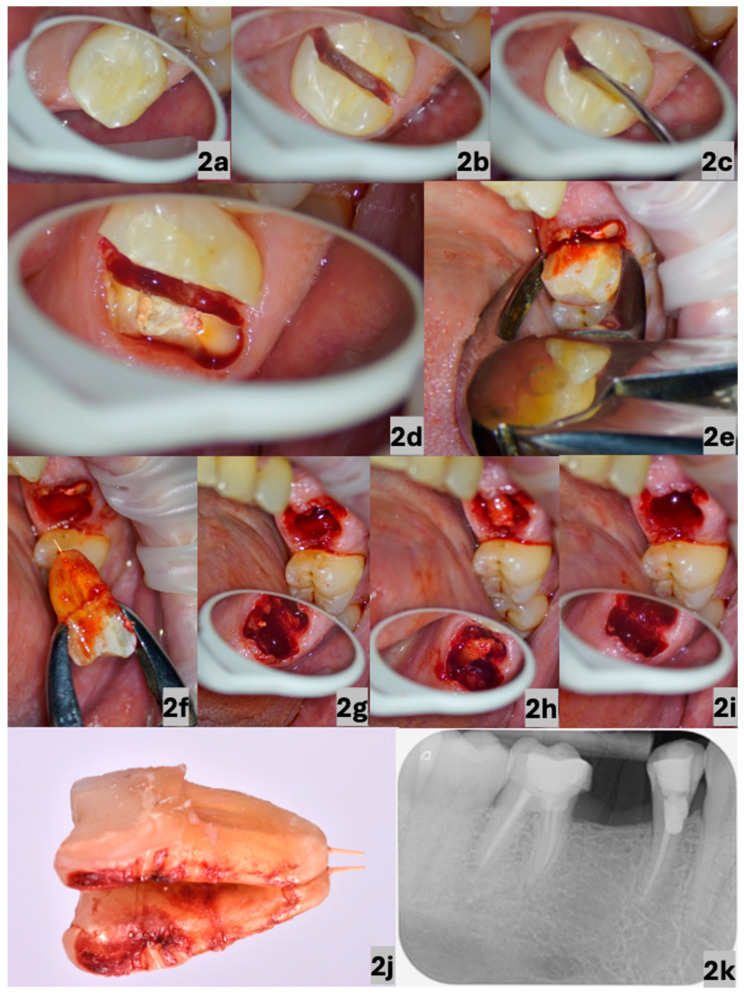
Occlusal view of the first right mandibular molar (**a**); root separation performed in the vestibulolingual direction in the centre of the pulp cavity floor (**b**); process of raising the roots with an elevator (**c**); fragmentation of the mesial part of the tooth (**d**); extraction of the distal root with root forceps (**e**); extracted distal root with visualised extruded gutta-percha cone (**f**); part of the mesial root, which was still present at the extraction site after repeated fragmentation (**g**); elevated mesial root at the extraction site (**h**); extraction site after elevation of the fragmented part of the root (**i**); detailed visualisation of the distal root with an extruded gutta-percha cone (**j**); preoperative intraoral radiograph of the first right mandibular molar; and the present periapical lesion with an extruded gutta-percha cone (**k**). The images in (**a**–**c**,**e**–**i**) were taken using an operating microscope under 6× magnification, and that in (**d**) was taken under 10× magnification.

A 54-year-old man was referred to the clinic for a consultation to assess the possibility of the re-endodontic treatment of the first right mandibular molar (Figure 2a). After considering complex therapy, which included re-endodontic treatment with the removal of the extruded gutta-percha cone (Figure 2j) from the distal root and subsequent prosthetic reconstruction, the patient opted for a tooth extraction.

The patient did not report any subjective complaints. His personal, medicinal, and allergic history was without clinical significance. Objectively, only the percussion sensitivity of the tooth was detected in the absence of other relevant symptoms, and the substandard quality of the previous endodontic treatment and a periapical lesion on the periapical radiograph were noted (Figure 2k).

Inferior alveolar nerve and buccal nerve block anaesthesia was performed using 1.7 mL of an articaine hydrochloride solution with adrenaline in a ratio of 1:100,000 (Septanest Forte, by Septodont, Saint-Maur-des-Fossés, France); afterwards, we chose to separate the roots prior to their elevation. Both of these procedures were performed using an operating CJ-Optik Flexion Twin microscope (CJ-Optik GmbH & Co. KG, Asslar-Werdorf, Germany) due to the increased risk of a tooth fracture during the extraction. The procedure included the separation of the roots (Figure 2b) without removing the coronal part. During the luxation of the distal and mesial roots (Figure 2c), a fracture of the mesial part of the crown occurred (Figure 2d). After the elevation of the distal root (Figure 2e–f), another fragmentation occurred when the elevation of the mesial root was attempted (Figure 2g). The situation required a reduction in the root volume to provide adequate access for elevator use. After the removal of all fragments (Figure 2h), a thorough debridement of the alveolar socket was performed. Considering the minimal traumatization of the surrounding tissues, it was not necessary to suture the extraction wound (Figure 2i). The patient was clinically re-evaluated 10 days postoperatively to assess soft tissue healing and to rule out complications such as dry socket or infection. A subsequent follow-up was conducted approximately 6 weeks after the procedure to confirm complete mucosal healing. Since no complications were observed, further radiographic follow-up was deemed unnecessary at that moment. Wound healing was considered uneventful, with no need for antibiotic therapy or the prolonged use of analgesics.

### 3.3. Case 3

**Figure 3:**
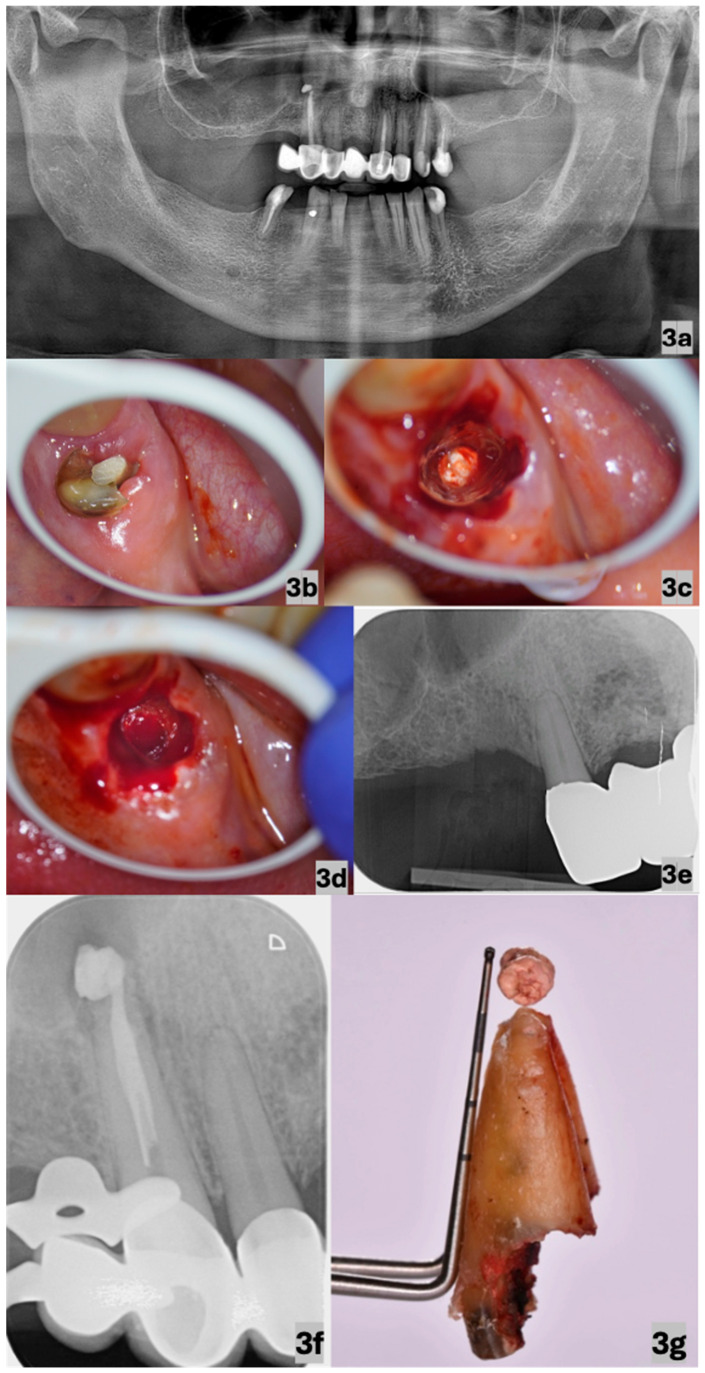
Panoramic radiograph (March 2023) showing the right maxillary canine with the present carious lesion and substandard endodontic treatment with extruded obturation material present (**a**); occlusal view of the decapitated right maxillary canine (**b**); visualisation of the extruded obturation material in the depth of the extraction wound after the extraction of the root (**c**); visualisation of the empty alveolar socket after the removal of the extruded endodontic materials (**d**); control periapical radiograph (February 2025)—clear evidence of successful bone healing was observed, with no periapical lesions or extruded materials present (**e**); periapical radiograph (October 2015) showing the extrusion of endodontic material during the obturation process (treatment performed by another dentist) (**f**); and detailed view of the extracted root with obturation material compared to the scale of a periodontal probe (**g**). The image in (**b**) was taken under 6× magnification and those in (**c**,**d**) were taken under 10× magnification using an operating microscope.

A 61-year-old man visited the clinic in September 2024 to extract the right maxillary canine (Figure 3b,g) due to the impossibility of its reconstruction. The extraction was part of a complex treatment plan.

Upon examination, the patient indicated the absence of subjective complaints, without the presence of a clinically significant personal, medicinal, or allergic history. Objectively, only the percussion sensitivity of the tooth was detected in the absence of other relevant symptoms, and the substandard quality of the endodontic treatment was shown on a panoramic radiograph and a perioperative periapical radiograph, with the significant extrusion of obturation materials (Figure 3a,f).

Under infiltration anaesthesia comprising 1.7 mL of an articaine hydrochloride solution with adrenaline in a ratio of 1:100,000 (Septanest Forte, by Septodont, Saint-Maur-des-Fossés, France), we proceeded to perform a partial separation due to the increased risk of a tooth fracture during the extraction, thereby gaining space for the manipulation with elevators and the subsequent luxation of individual fragments using an operating CJ-Optik Flexion Twin microscope (CJ-Optik GmbH & Co. KG, Asslar-Werdorf, Germany). After the elevation of all the fragments, the extruded material that had to be removed was still present in the alveolar socket (Figure 3c). Using superior magnification and maximum illumination, a thorough debridement of the alveolar socket was performed, with the successful removal of the extruded materials (Figure 3d). Considering the minimal traumatization of the surrounding tissues, it was not necessary to suture the extraction wound. The patient was clinically re-evaluated 7 days postoperatively to assess soft tissue healing and to rule out complications such as dry socket or infection. A subsequent follow-up was conducted approximately 4 weeks after the procedure to confirm complete mucosal healing. Since no complications were observed, further radiographic follow-up was deemed unnecessary at that moment.

Wound healing was considered uneventful, with no need for antibiotic therapy or prolonged analgesic use. The control periapical radiograph in February 2025 showed clear evidence of successful bone healing, with no periapical lesions or extruded materials present (Figure 3e).

## 4. Discussion

The aim of this case report was to expand upon the existing scarce literary data and to further demonstrate the advantages of the use of a microscope in dentoalveolar surgery. The clinical outcomes of the patients in this case report demonstrate the overall benefit of the use of an operating microscope during tooth extraction. Our findings are consistent with the literary data, which are so far very scarce. Mamoun [9] found that the use of microscope-level magnification combined with coaxial, head-mounted illumination was very useful when extracting teeth, and concluded that it enables dentists to detect tooth particles and their respective perimeters more systematically. In a recent randomised controlled study [8], thirty-three patients were allocated to two groups and treated with the use of either dental loupes or an operating microscope to study alveolar ridge protection. The microscope-assisted procedure was associated with a significantly higher chance of removing granulomatous tissue, favourable early healing, and a similar crestal bone quality. All the documented advantages and possible disadvantages of using an operating microscope are discussed below.

### 4.1. Minimal Invasiveness

Compared to conventional procedures, minimally invasive techniques are generally better tolerated by patients [10]. A lower invasiveness reduces the risk of complications due to tissue trauma and is associated with a shorter recovery [11], as was the case with the patients presented in this article. The conventional approach to tooth extraction involves raising the mucoperiosteal flap and performing significant alveolar bone preparation. A minimally invasive procedure with the use of an operating microscope allows access to the root of the tooth directly through the alveolar socket without the need for radical preparation, especially of the vestibular alveolar bone. An operating microscope enables access and visualisation while at the same time eliminating the need to raise the mucoperiosteal flap [11].

### 4.2. Root Separation

When extracting multi-rooted teeth, there is an increased risk of breaking one or more roots, especially if the separation of the roots is not performed prior to the final elevation. A root fracture is a complication that usually prolongs the procedure. A conventional approach alters the planned simple extraction as it requires significant bone preparation, flap elevation, and subsequent suturing [12]. Intense magnification and illumination enable the detailed differentiation of individual tooth structures, such as the entrances to root canals, and help guide separation in the right direction. Multi-rooted teeth, especially upper molars, may be rotated or have a nonstandard root anatomy. These factors can lead to incorrect separation, which greatly complicates the procedure; thus, the extraction can become more complicated than it was before the root separation was attempted. Separating teeth using high magnification also helps prevent iatrogenic damage to adjacent tissues. Single-rooted teeth can also be separated, with the separation being carried out vertically in the root space. In this way, space is created for elevator use, and the need for alveolar bone preparation around the tooth is eliminated.

### 4.3. Atraumatic Extraction

In the literature, the term “atraumatic extraction” refers to minimally invasive procedures that can be used to extract teeth, thereby reducing iatrogenic traumatization [11]. When extracting the roots one by one instead of all at once, a smaller luxation force is sufficient; this reduces the traumatization of the surrounding tissues, the risk of breaking off the vestibular alveolar bone (the bundle bone of the alveolar bone), damage to the neighbouring teeth, and other complications related to tooth extractions.

Traumatization and the use of excessive luxation forces are two of the main factors in the development of dry socket, which can be accompanied by secondary wound healing and intense pain [13]. A less invasive procedure is associated with fewer complications and, as a rule, with a more favourable course of healing [8]. Possible post-procedure inflammation and pain are also reduced and are also associated with a reduced need for antibiotics [14] and/or analgesics [15]. Different extraction systems have also been developed for atraumatic extractions [16].

### 4.4. Alveolar Bone and Soft Tissue Healing After Extraction

After a standard tooth extraction, the alveolar ridge atrophies. Within one year after extraction, the width of the alveolus is resorbed by an average of 50% [17]. This resorption is even more significant when the vestibular lamella of the alveolar bone is damaged, which is one of the most endangered anatomical structures during a tooth extraction. Its preservation reduces the need for and extent of subsequent alveolar bone augmentation [18].

Teeth indicated for extraction often present with periapical lesions. The presence of the remnants of periapical granulomatous tissues can interfere with the quality of bone healing. The use of an operating microscope provides a significantly higher chance of completely removing granulomatous tissues compared to the use of dental loupes [9].

Gentle extraction in conjunction with root separation reduces the risk of damaging the vestibular bone. The resorption of the alveolar process takes place after each tooth extraction; however, the need to raise the mucoperiosteal flap during a conventional approach to a broken fragment increases the rate of the resorption of the alveolus [19]. The incision associated with the opening of the mucoperiosteal flap can result in the formation of scars in the aesthetic area or recesses around the adjacent teeth. Damage to sensitive anatomical structures such as the mental nerve can occur while manipulating instruments in the apical region of the lower second premolars [20]. For these reasons, the authors deviate from the use of conventional approaches to fragmented tooth roots and prefer the direct visualisation of the alveolar socket through the socket itself.

### 4.5. Limitations of Operating Microscope Use

Although rarely discussed in the literature, some challenges arise with microscope use, especially in dentoalveolar surgery. Higher magnification levels limit the depth and width of the field, while lower magnification levels, such as 6–10×, are more practical for extractions. Working with indirect vision means that one hand is constantly occupied with a mirror. This requires strong teamwork and places increasing demands on coordination. Mirror contamination (saliva, blood) and restricted access to distal areas (e.g., the lower wisdom teeth) may also complicate the use of a microscope. These problems are encountered mainly by dentists with insufficient experience in using an operating microscope, especially during surgical procedures [21]. Suturing can be challenging under an indirect view, and therefore, loupes are preferred by the authors for this. Some patients may not tolerate the reclined position required for optimal ergonomics, while some physicians may consider the initial costs of an operating microscope to be significantly high, and some may perceive a prolonged static posture without breaks as a physical strain [22]. Despite the above, microscope use improves precision, visibility, and ergonomics and supports minimally traumatic extractions.

Taken together, the scarce literature [8,9] and clinical data suggest that the use of an operating microscope during teeth extractions makes it possible to reduce trauma to the surrounding tissues and to decrease post-procedural complications and the need for antibiotics and analgesics. Minimising the traumatization of the alveolar bone and soft tissues creates optimal conditions for the subsequent insertion of an implant, often eliminating the need for more complex augmentation procedures.

A significant advantage of the use of an operating microscope is its ability to provide adequate magnification and intense coaxial illumination, substantially improving the overview in the operating field. A better overview enables the detailed visualisation and precise identification of individual structures, which supports the precise execution of extraction procedures. At the same time, a strong light source and magnification reduce eye strain [3]. Treatments can be performed with the minimal movement of the dentist, reducing the physical burden [23]. Moreover, treatments performed with an operating microscope seem to be better accepted by patients, and the use of a microscope enhances their comfort during the procedure [24].

## 5. Conclusions

This case report, by achieving primary and secondary endpoints, demonstrates the overall benefits of using an operating microscope during tooth extractions in terms of the clinical outcomes of patients. This tool represents a step towards the more modern and efficient provision of dental care, which reflects the current trends toward minimising invasiveness and maximising patient care. For clear evidence-based confirmation of the clinical benefit, comparative studies with larger samples of patients are needed.

## Data Availability

The original contributions presented in this study are included in the article. Further inquiries can be directed to the corresponding author.
